# Demographic and occupational profile of dietitians working in dialysis centers in Brazil

**DOI:** 10.1590/2175-8239-JBN-2023-0017en

**Published:** 2023-07-17

**Authors:** Fabiana Baggio Nerbass, Aline de Araujo Antunes, Lilian Cuppari

**Affiliations:** 1 Fundação Pró-Rim, Joinville, SC, Brazil.; 2 Sociedade Brasileira de Nefrologia, Comitê de Nutrição, São Paulo, SP, Brazil.; 3 Universidade Federal de São Paulo, São Paulo, SP, Brazil.

**Keywords:** Dietitians, Nutrition, Dialysis, Nutricionista, Nutrição, Diálise

## Abstract

**Introduction:**

In 2004, the Ministry of Health stipulated that dialysis centers were required to have at least one dietitian on their staff. However, the regulation did not include recommendations regarding the number of dietitians or the workload based on the number of patients assisted.

**Objective:**

To describe the demographic and occupational profiles of dietitians working in dialysis centers in Brazil.

**Methodology:**

An electronic questionnaire was disseminated in social media and messaging apps with questions about the demographic and occupational profile of dietitians working in dialysis centers and matters related to patient care.

**Results:**

A total of 207 questionnaires were answered, covering 24% of the dialysis centers in Brazil. More than half of the dietitians (58%) had worked for more than five years in dialysis centers, and 83% reported additional training in Nephrology. The median (interquartile range) number of patients per monthly working hour was 1.6 (1.0-2.3). Considering all dialysis centers, 64% of the patients were seen at least once a month. Differences in demographic/occupational profiles and patient care were associated with workload, the main source of dialysis funding, and Brazilian geographical region.

**Conclusion:**

Most dietitians were experienced and trained in Nephrology. Substantial variability was found in the number of patients per dietitian workload, and proportion of patients receiving monthly nutritional care. Further studies are needed to discuss the demands of dietitians, dialysis centers, and patients.

## RESUMO

**Introdução:**

Em 2004, o Ministério da Saúde estabeleceu que cada serviço de diálise deve ter no mínimo um nutricionista vinculado a ele. Porém, a regulamentação não incluiu recomendações em relação ao número de profissionais ou à carga horária de acordo com o número de pacientes assistidos.

**Objetivo:**

Conhecer o perfil e as práticas de nutricionistas que atuam em unidades de diálise brasileiras.

**Metodologia:**

O questionário eletrônico divulgado em mídias sociais e aplicativos de mensagens incluiu questões que abrangiam características do perfil demográfico e ocupacional do profissional e da unidade de diálise, além de perguntas relacionadas ao atendimento dos pacientes.

**Resultados:**

Foram recebidos eletronicamente 207 questionários, o equivalente a 24% das unidades de diálise brasileiras. Mais da metade dos nutricionistas (58%) atuava havia mais de cinco anos em unidades de diálise e 83% referiram formação complementar na área da Nefrologia. A mediana (interquartis) do número de pacientes por hora mensal de trabalho foi 1,6 (1,0-2,3). Considerando todas as unidades, o percentual de pacientes atendidos mensalmente foi correspondente a 64%. Diferenças no perfil e nas práticas foram encontradas de acordo com a carga horária, principal fonte financiadora da unidade de diálise e região demográfica brasileira.

**Conclusão:**

A maioria dos nutricionistas tem boa experiência e formação na área. Foi encontrada uma grande variabilidade em relação ao número de pacientes por carga horária do profissional e do percentual de indivíduos que recebiam atendimento nutricional mensal. São necessárias investigações que avaliem questões relacionadas tanto às demandas dos profissionais quanto às das unidades contratantes e dos pacientes.

## Introduction

Individuals with chronic kidney disease (CKD), especially on dialysis, are at increased risk for nutritional and metabolic disorders that may compromise the quality of life, increase the cost of care, and the risk of death. Thus, as recommended in the clinical practice guideline on nutrition for patients with CKD (Kidney Disease Outcomes Quality Initiative – KDOQI, 2020), a dietitian should assess and monitor individuals with this disease^1^.

Resolution 154 of the Brazilian Ministry of Health, issued on June 15, 2004, established the technical grounds for the operation of dialysis centers and required each unit to have at least one dietitian on its staff^2^. However, the resolution did not include recommendations regarding the number of dietitians or the workload based on the number of patients, as has been the case for other professionals in the multidisciplinary care team.

Eighteen years after the enactment of this regulation, little is known about the profile and patient care practices of dietitians in Brazilian dialysis centers. Therefore, we developed an electronic questionnaire to uncover the demographic characteristics of dietitians and the dialysis units where they work and the patient care practices used in routine nutritional care. This article describes and discusses dietitians’ demographic and occupational profile and the dialysis centers where they work.

## Methods

The questionnaire formulated by members of the Nutrition Committee of the Brazilian Society of Nephrology (BSN) and other dietitians with a track record in nephrology was shared in the social media platforms of the BSN and dietitian messaging groups in September 2022. Respondents were allowed to remain anonymous and were not required to inform the dialysis center where they worked. Dietitians working in multiple dialysis centers were instructed to fill out separate forms for each clinic.

The questionnaire developed on Google Forms contained questions that covered elements pertaining to the respondents’ professional background (years after graduation, years working at dialysis centers, additional training in renal nutrition), occupation (employment arrangement, compensation, workload), and dialysis center (Brazilian State, number of patients, funding). About patient care, participants were asked about the number of patients on dialysis they saw monthly, delivery of care (on-demand or pre-established schedule), and other assignments besides providing care to patients on dialysis (providing care to renal transplant patients or patients on conservative management, managing the food service).

We calculated the proportion of patients seen monthly and by subcategories. The ratio between the number of patients (N) and the monthly workload was obtained by dividing N by the weekly workload multiplied by five. E.g.: In a unit with 120 patients on dialysis in which a dietitian works for 20 hours a week, the number of patients per hour per month is 1.2 (120/100). Units staffed with civil servants (N = 12) were excluded from the calculation because their workload includes other assignments outside the dialysis unit.

## Statistical Analysis

Statistical analysis was performed on SPSS version 21.0 for Windows (SPSS, Inc. Chicago, IL, USA). Results were presented as proportions, median values, and interquartile ranges when appropriate. The variables between groups were compared using the chi-squared test for categorical variables and the Mann-Whitney or the Kruskal-Wallis test complemented by Dunn’s test for continuous variables. Statistical significance was considered for p-values < 0.05.

## Results

A total of 207 questionnaires were answered by 202 dietitians (one worked in three and two in two dialysis units), covering 24% of the 849 active dialysis centers registered with the BSN.^3^ The geographic regions with the highest percent participation were the Northeast and Midwest (28%), followed by the South and North, 27% each, and the Southeast region, with 21% of the participating clinics.

Regarding the dialysis funding, 57% of the dialysis centers were predominantly and 20% solely funded by the Brazilian public healthcare system (SUS – Sistema Único de Saúde). Most patients in 8% and all patients in 15% of the dialysis centers were funded by private health insurance.

Only three dialysis centers did not offer meals/snacks to patients on hemodialysis (HD). Most offered food during dialysis sessions (72%), 10% prior to sessions, 8% after sessions, and 10% offered food on more than one occasion.

The median (interquartile range) number of patients on HD per dialysis center was 191 (120-262). Of the 207 units, 116 (56%) also offered peritoneal dialysis (PD); the median number of patients on PD was 15 (4-40). Considering both types, the units had 200 (129-300) patients on dialysis. Regarding routine nutrition care, 41% of the units had pre-established schedule (e.g.: once a month, once every three months), 21% nutrition care on demand whenever requested by other team member, and 38% both periodically or on demand.

Table 1 contains the answers to the questions related to the demographic and occupational profile of the dietitians. More than half (58%) had worked for more than five years in dialysis units, and only 17% had no additional specific training in nephrology.

**Table 1.** Demographic and occupational characteristics of dietitians from participating dialysis centers

*Time since graduation* ≤ 5 years 6 to 10 years 11 to 15 years > 15 years	20% 23% 24% 33%
*Time working in dialysis centers* < 2 years 3 to 4 years 5 to 10 years > 10 years	27% 14% 28% 30%
*Additional training in nephrology* None Capacity Building Specialization Master’s and/or Doctoral Degree	17% 30% 43% 10%
*Weekly working hours at the dialysis unit < 20 hours 20 to 30 hours > 30 hours	14% 54% 32%
*Employment arrangement* Consolidated Labor Laws Business contractor Individual contractor Civil servant	80% 6% 8% 6%
*Wage in minimum salaries (MS) < 1 MS 1 to 2 MS 2 to 4 MS 4 to 6 MS > 6 MS	4% 47% 43% 5% 1%
*Assignment in addition to dialysis patients care* Conservative management patients Kidney transplantation patients Food service management	36% 16% 59%

* Dietitians who are civil servants were excluded since their working hours and salaries do not include only work at a dialysis center.

Most (53%) worked between 20 and 30 hours a week, and 80% were hired as stipulated in the Brazilian Consolidated Labor Laws. Half (51%) declared receiving compensation of up to two minimum wages (a minimum wage in September 2022: BRL 1,212.00). In addition of the patients care on dialysis, more than half (59%) of the respondents were also responsible for managing the food service of the unit, more than a third (36%) for the care of patients on conservative treatment (36%), and 16% for the care of kidney transplant patients.

**Figure 1.**
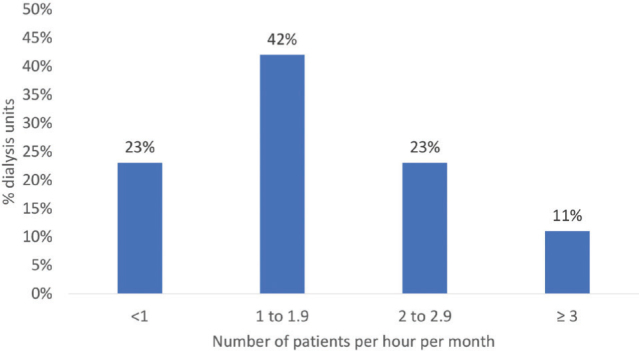
Number of patients on dialysis per hour per month of the dietitians working in dialysis centers.

The median (interquartile range) number of patients per hour per month was 1.6 (1.0-2.3). The distribution of dietitians into four categories of this metric (< 1 to ≥ 3 patients/hour/month) is presented in Figure 1. Sixty-four percent of the patients from all dialysis units were seen monthly by a dietitian. When the group of patients seen monthly was categorized into tertiles (≤ 33%; 34 to 66%; ≥ 67% of the total), we found that in 21% of the units, less than a third of the patients were seen monthly; in 24% of the units, between one and two-thirds were seen monthly; and in the remaining 55% of the units, more than two-thirds received nutritional care every month.

**Figure 2.**
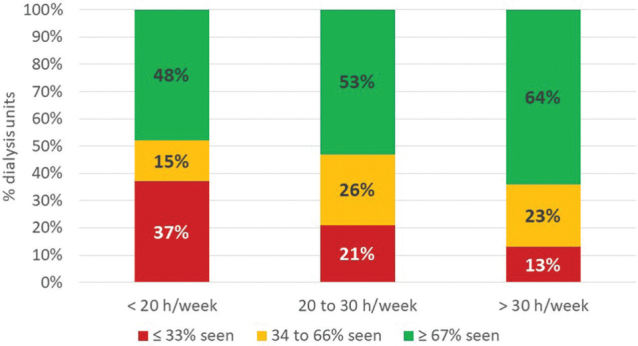
Proportion of patients seen monthly by dietitians based on weekly working hours.

Table 2 shows the comparisons between numbers and proportions of patients seen monthly in the dialysis units according to dietitian workload. The higher the workload, the greater the total number and mean proportion of patients seen monthly by a dietitian. When the proportions of patients seen monthly by a dietitian were categorized into tertiles (Figure 2), we found that the lower the workload, the higher the proportion of clinics in which less than a third of the patients were seen monthly (37% with workload < 20h/week, 21% with workload of 20 to 30 h/week; and 13% with > 30 h/week).

**Table 2.** Number of patients in dialysis units and proportion of patients per category of weekly workload

Weekly workload	Number of patients	Proportion of patients seen monthly
< 20 hours	176 (75-200)	56%
20 to 30 hours	215 (150-270)	66%
> 30 hours	240 (140-350)	68%
Total	200 (129-300)	64%

Median (interquartile range).

**Figure 3.**
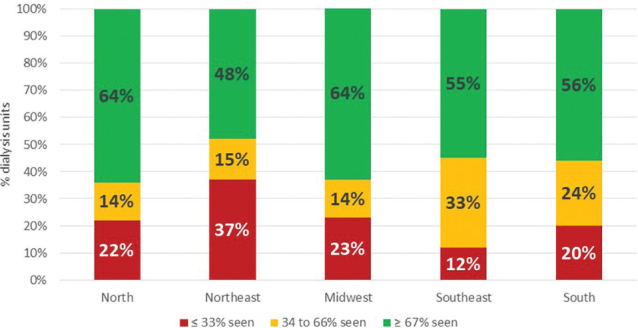
Proportion of patients on dialysis seen monthly based on demographic region.

In the analysis by demographic regions, dietitians in the Northeast had a significantly greater number of patients per hour per month than their counterparts in the North, Southeast, and South regions. The proportion of patients seen monthly ranged from 55% in the Northeast to 73% in the North region (Table 3). In addition, the categorization of patients seen monthly in tertiles (Figure 3) showed that in the North and Midwest regions, almost two-thirds of them were seen monthly (64%), a higher proportion than in the other regions of Brazil.

**Table 3.** Number of patients per workload and proportion of patients seen monthly in each demographic region

Demographic region	N per hour per month	% seen monthly
North	1.20 (0.78-1.71)	73%
Northeast	2.17 (1.44-2.92)^a,b,c^	55%
Midwest	1.34 (0.53-1.81)	62%
Southeast	1.57 (1.18-2.17)	67%
South	1.40 (0.81-2.10)	66%

^a^P < 0.05 *versus* North; ^b^P < 0.05 *versus* Southeast; ^c^P < 0.05 *versus* South.

Table 4 shows the data regarding the payment/reimbursement for dialysis treatment. The number of patients on dialysis and the number of patients seen per hour were higher in dialysis centers funded predominantly or exclusively from the Brazilian public healthcare system. The proportion of patients seen monthly was similar in both groups. In terms of assignments other than providing care to patients on dialysis, dietitians working in clinics where care is funded mainly via the public healthcare system were more frequently involved in the care of patients on conservative treatment and less in the management of food services, than their counterparts working in clinics in which care is funded by other means.

**Table 4.** Comparisons between units based on source of payment/reimbursement as primarily or exclusively public or private

	Public (N = 160)	Private (N = 47)	P
*Patients o dialysis* Number Number/hour per month % seen monthly	209 (144-304) 1.8 (1.1-2.4) 63%	146 (80-264) 1.2 (0.6-1.7) 67%	0.006 < 0.001 –
*Other assignments (% dietitians)* Conservative management Kidney transplant patients Food service management	39% 17% 56%	25% 11% 74%	0.02 0.26 0.02

Median (interquartile range).

## Discussion

This is the first investigation developed in Brazil that studied the demographic and occupational characteristics of dietitians working in dialysis centers.

Most of the respondents graduated in Nutrition for more than ten years; had worked for more than five years in dialysis centers; and had complementary training in nephrology. We found only one paper describing dietitians working in nephrology. In an online questionnaire answered by 599 dietitians affiliated with international organizations (91% from USA), 77% had worked in nephrology for at least six years^4^.

However, it is important to understand that the profile described in this study, which covered about a quarter of the dietitians working in dialysis units, probably do not faithfully reflect the national reality, since the survey was disseminated primarily via organized professional messaging applications, in addition to the social media platforms of the BSN. Although dialysis centers are required to have dietitians on their staff, they are not required to register them as technical leads with the board of dietitians, differently from other members of the staff such as physicians and nurses, which limited our access to dietitians and the reach of our study.

Important variations were observed in the delivery of care (periodic or on demand), as well as in the number of patients on dialysis per monthly workload. These fluctuations reflect the lack of occupational regulations, leaving the matter in the hands of their employers, independent of the dialysis unit demand. Although these numbers give us an idea of the amount of time dietitians for each patient, they do not consider the time spent on other assignments, such as organizing events, preparing orientation materials, and filling up medical records. Most participants are also responsible for managing food services in the clinic and providing care to patients with different stages of CKD. Resolution 600/2018 of the Federal Board of Dietitians, a regulation that stipulates the minimum parameters needed for dietitians to provide effective care, recommends a dietitian for renal replacement therapy services with a workload of 30h/week for every 50 patients/day, that is, a ratio of 0.3 patient per hour per month. The recommendations also stipulate that the size of the team of dietitians should be calculated based on the outpatient care needs of the unit and the number of patients seen regularly at the dialysis center^5^. However, this recommendation was not based on studies and is far from representing the reality seen in Brazil.

Another relevant variation was observed in the proportion of patients seen monthly. While less than a third of the patients received nutritional care monthly in a fifth of the units, in 30% of them all patients received monthly care. It is important to note that the question did not define what constitutes care, which may vary between respondents.

The number of working hours affected the proportion of patients seen monthly. A higher proportion of patients did not receive monthly care in units where dietitians work for less than 20 hours a week compared to units where they work more hours.

Differences were also seen between demographic regions. Dietitians working in the Northeast had more patients per hour per month than in other three regions (North, Southeast and South). This cannot be explained by the fact that the region has a lower proportion of privately funded centers (17%), since the proportion was not significantly different from that of other regions, which ranged from 14% (South) to 36% (North). We were unable to ascribe a reason for this finding.

With regard to the payment/reimbursement for dialysis treatment, although the proportion of patients seen monthly was similar between the two groups (public healthcare system vs. private health insurance), dietitians working in units in which payment was received mainly or exclusively from private health insurance had fewer patients per hour per month and, therefore, spent more time with each patient.

According to Ordinance 389/2014 of the Ministry of Health, dialysis units must offer, under the guidance of a dietitian and based on medical prescription, nutritional support to patients on the day of dialysis^6^. Almost all dialysis units offer patients a meal/snacks, and most do so during dialysis sessions. As recommended by the International Society of Renal Nutrition and Metabolism, this practice may improve one’s nutritional status, reduce inflammation, increase patient satisfaction, and may improve quality of life related to health and survival^7^. Since there is no legal definition the nutritional composition of what is offered to patients, it might be interesting investigate the nutritional quality of the food items served to patients.

A quarter of the dietitians working in dialysis centers in Brazil participated in our study. The results indicated the existence of significant variations in the number of patients seen per hour per month and in the proportion of patients receiving monthly care in dialysis units. Additional studies are needed to establish recommendations about the role of dietitians in dialysis services, taking into account the needs of dietitians, employers, and patients on dialysis, in order to ensure the proper delivery of nutritional care and support. It is also essential that the public healthcare system, which funds the dialysis treatment of most patients in Brazil, increases the fees paid for dialysis care, to make it feasible to hire dietitians and other professionals on the multidisciplinary team with adequate remuneration and workload for optimal service delivery, to ultimately improve the quality of care.
